# Follicular dendritic-like cells derived from human monocytes

**DOI:** 10.1186/1471-2172-6-23

**Published:** 2005-09-22

**Authors:** Dagmar EH Heinemann, J Hinrich Peters

**Affiliations:** 1 Max-Planck-Institut für Biophysikalische Chemie, D-37077 Goettingen, Germany; 2 Abteilung für Immunologie, D-37075 Göttingen, Germany

## Abstract

**Background:**

Follicular dendritic cells (FDCs) play a central role in controlling B-cell response maturation, isotype switching and the maintenance of B-cell memory. These functions are based on prolonged preservation of antigen and its presentation in its native form by FDCs. However, when entrapping entire pathogens, FDCs can turn into dangerous long-term reservoirs that may preserve viruses or prions in highly infectious form.

Despite various efforts, the ontogeny of FDCs has remained elusive. They have been proposed to derive either from bone marrow stromal cells, myeloid cells or local mesenchymal precursors. Still, differentiating FDCs from their precursors *in vitro *may allow addressing many unsolved issues associated with the (patho-) biology of these important antigen-presenting cells. The aim of our study was to demonstrate that FDC-like cells can be deduced from monocytes, and to develop a protocol in order to quantitatively generate them *in vitro*.

**Results:**

Employing highly purified human monocytes as a starter population, low concentrations of Il-4 (25 U/ml) and GM-CSF (3 U/ml) in combination with Dexamethasone (Dex) (0.5 μM) in serum-free medium trigger the differentiation into FDC-like cells. After transient *de-novo *membrane expression of alkaline phosphatase (AP), such cells highly up-regulate surface expression of complement receptor I (CD35). Co-expression of CD68 confirms the monocytic origin of both, AP^pos ^and CD35^pos ^cells. The common leukocyte antigen CD45 is strongly down-regulated. Successive stimulation with TNF-α up-regulates adhesion molecules ICAM-1 (CD54) and VCAM (CD106). Importantly, both, AP^pos ^as well as AP^neg ^FDC-like cells, heterotypically cluster with and emperipolese B cells and exhibit the FDC characteristic ability to entrap functionally preserved antigen for prolonged times. Identical characteristics are found in monocytes which were highly expanded *in vitro *by higher doses of GM-CSF (25 U/ml) in the absence of Dex and Il-4 before employing the above differentiation cocktail.

**Conclusion:**

In this work we provide evidence that FDC-like cells can be derived from monocytes *in vitro*. Monocyte-derived FDC-like cells quantitatively produced offer a broad utility covering basic research as well as clinical application.

## Background

Until today, the ontogeny of follicular dendritic cells (FDCs) has remained unresolved [1,2]. Even transplantation experiments have led to contradictory results [3-11]. Researchers contemplating a bone marrow origin have suggested that these professional antigen-presenting cells derive either from the lymphoid [5] or the myeloid lineages [10]; from monocytes in particular [12,13]; or even from stromal cells of the bone marrow [6,11,14,15]. Alternatively, FDCs have been claimed to originate within lymphoid organs from local mesenchymal precursors [6,7,11,14,16-18]. So far, however, no protocol is available to generate FDCs *in vitro *from their putative progenitors [2].

The monocyte is increasingly acknowledged as the cornerstone of a plastic differentiation system. Accordingly, the findings of a monocytic origin of both macrophages [19] and dendritic cells (DCs) [20]; (for review see [21]) have subsequently been complemented by evidence proving that microglia [22] and osteoclasts [23] can be derived from this cell type as well. More recent results have revealed that even osteoblasts [24], neural cells, hepatocytes [25] and endothelial cells [26] can be generated from certain monocyte subsets. Therefore, its remarkable developmental capacity strongly indicates that the monocyte actually represents a somatic stem cell.

Our previous findings demonstrated that the bone/liver/kidney isozyme of Alkaline Phosphatase (AP) is likewise expressed in cells obtained from *ex vivo*-explanted foreign-body granuloma as well as in monocyte-derived *in-vitro *granuloma. Co-expression of CD68 assigned such AP^pos ^cells to the myeloid lineage. Intriguingly, cultured osteoblasts were found to be phagocytic and co-expressed AP and CD68. Taken together, these results strongly implied osteoblasts and macrophages to derive from a common source [27,28]. AP of the bone/liver/kidney isozyme is also expressed by osteoblasts, activated endothelial cells, stromal reticulum cells [29]) as well as by FDCs [30]. Consequently, we hypothesized that AP expression may constitute a link between monocytes and mesenchymal cells [27]. Indeed, when attempting to identify signals provoking AP expression in monocytes, we found it transiently up-regulated in GM-CSF-induced proliferating monocyte cultures. AP expression was further enhanced and prolonged by IL-4. In the absence of IL-4, AP was down-regulated, while typical osteoclast markers such as tartrate-resistant acid phosphatase and vitronectin receptor, were expressed [27].

Our present study aimed at determining whether FDCs can be derived from the monocytic lineage. As a result, we present evidence that under defined serum-free conditions highly purified human monocytes differentiate into functionally competent FDC-like cells. Low concentrations of Il-4 and GM-CSF in combination with Dexamethasone (Dex) were used to induce AP expression as a striking feature of these cultures accompanied by some of the most important features of FDCs, expression of CD35 [31,32], B cell rosetting and emperipolesis [33], as well as antigen trapping and retaining it for prolonged time [34].

## Results

### Generation and phenotyping of monocyte-derived primary FDC-like cells

**Figure 1:**
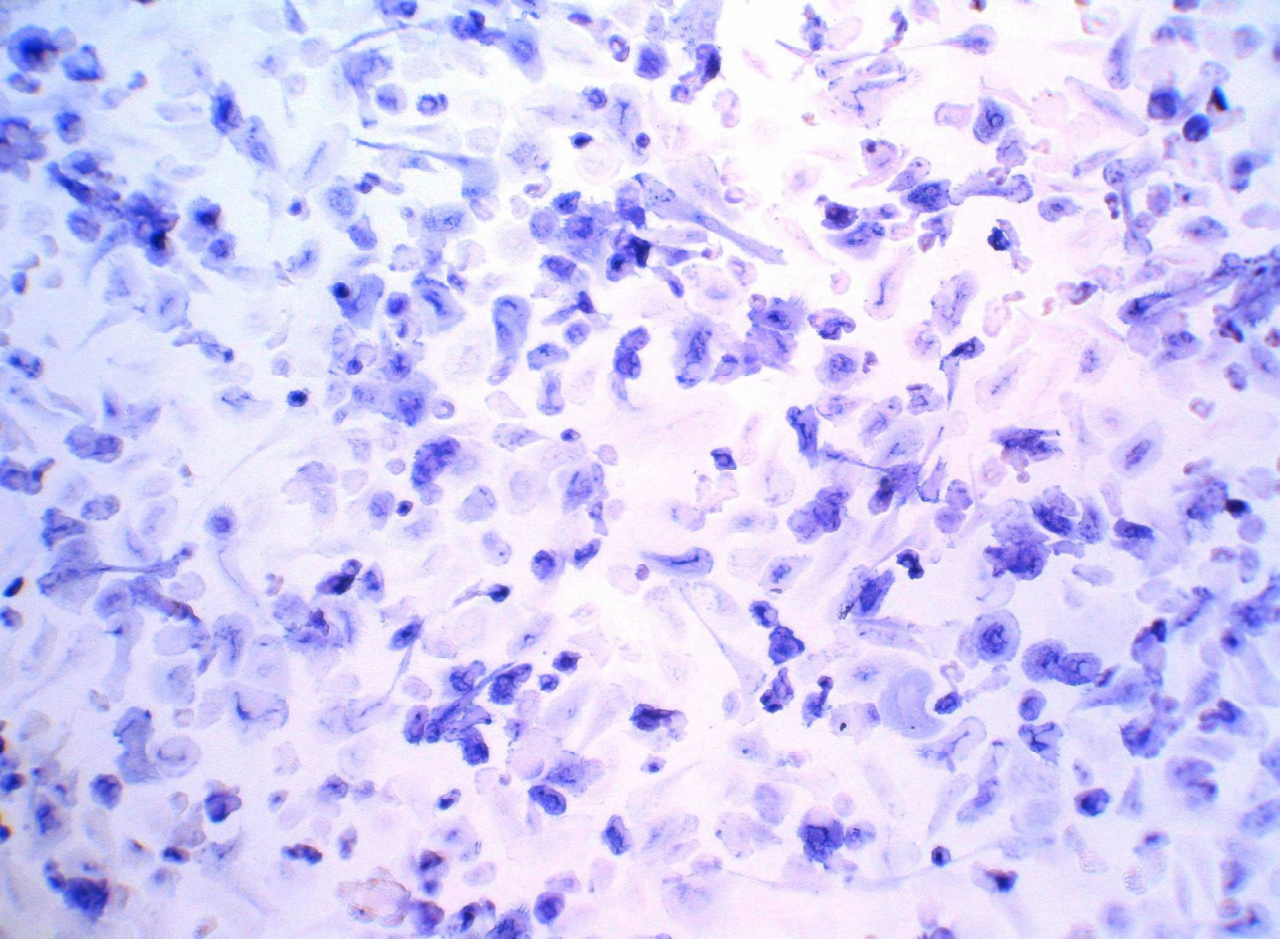
**AP expression in FDC-like cells**. After 15 days of culture the presence of Il-4 (25 U/ml), GM-CSF (3 U/ml) and Dex (0, 5 μM), monocyte-derived cells were fixed and stained for AP (blue).

**Figure 2:**
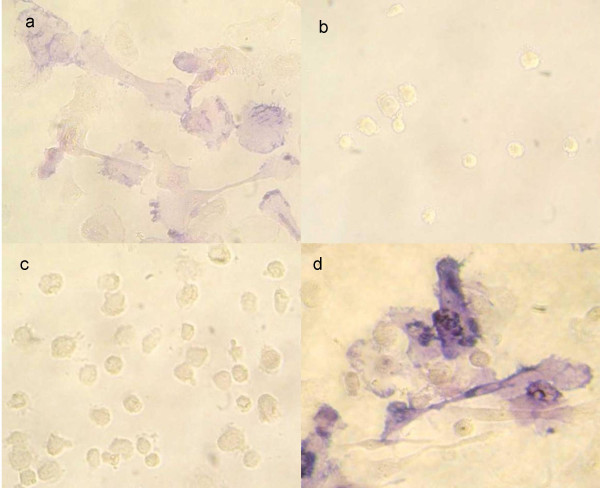
**Synergistic action of AP-inducing factors**. Monocytes were cultured in the presence of **a**, 25 U/ml Il-4 and 3 U/ml GM-CSF; **b**, 0, 5 μM Dex; **c**, 3 U/ml GM-CSF; **d**, 25 U/ml Il-4, 3 U/ml GM-CSF and 0, 5 μM Dex (full mix). On day 15, the cultures were fixed and stained for expression of AP.

Highly enriched human monocytes highly depleted of lymphocytes and cultured for 12 or 15 days in the presence of IL-4 (25 U/ml), GM-CSF (3 U/ml) and Dex (0,5 μM), tightly adhered to the substratum, exhibited multiple shapes as well as numerous projections and thrived closely adjacent to one another. Typically, these cell variants strongly expressed AP (Fig. 1), with a peak on day 12. These cells were referred to as primary FDC-like cells. For a maximal expression of AP the three inducers had to be present to act synergistically, whereas GM-CSF plus IL-4 in the absence of Dex induced expression of AP to a lesser extent only (Fig. 2a–d). IL-4 could be replaced by IL-13 (100 U/ml) (not demonstrated).

**Figure 3:**
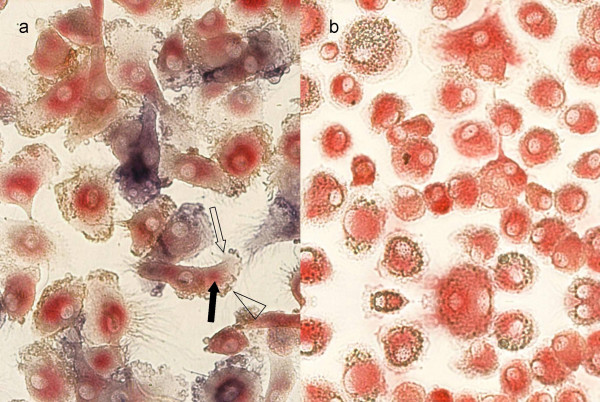
**AP^pos ^cells express the FDC-marker CD35 and the myeloid marker CD68**. Monocytes were cultured under conditions described in Fig. 1 for differentiating into FDC-like cells (**a**) or for macrophages (**b**) and triple-stained for AP, CD 35 and CD68. **a**, FDC-like cells co-expressing CD68 (red; filled arrow), AP (blue; open arrow), and CD35 (brown; arrowhead) at different degrees; **b**, control cells cultured in parallel in the presence of macrophage differentiation conditions stained for AP (negative), CD35 (negative), CD68 (positive, red).

By triple-staining we found AP^pos ^and AP^neg ^cells expressing membrane-bound CD35 (complement receptor I) (Fig. 3a). Both, AP^pos ^as well as CD35^pos ^cells, could further be co-stained for the monocyte/macrophage marker CD68, thus revealing the myeloid origin of the cells (Fig. 3a). For control, AP expression was virtually absent, and CD35 was only occasionally seen in parallel cultures where monocytes had been induced to differentiate into macrophages (Fig. 3b).

**Figure 4:**
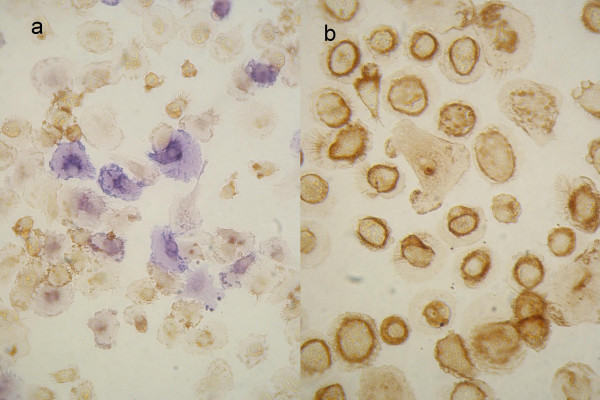
**Down-regulation of CD45 in FDC-like cells**. Co-staining for AP and CD45 in FDC-like cells or macrophages. **a**, CD45 (brown) is down-regulated in FDC-like AP-positive (blue) cells compared with **b**, macrophages as controls where CD45 is fully expressed, and AP is negative.

The common leukocyte marker CD45 was strongly down-regulated in FDC-like cells, obviously correlating with AP expression (Fig. 4a) as compared with constitutively CD45^pos ^macrophages (Fig. 4b).

### B-cell rosetting and emperipolesis

**Figure 5:**
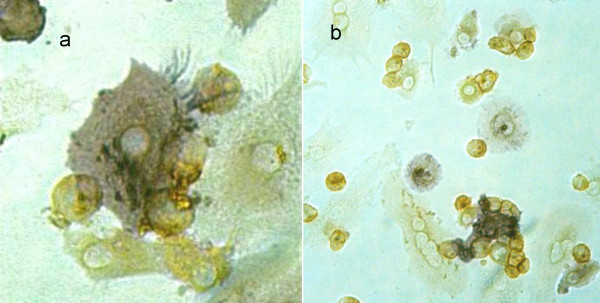
**Heterotypic B-cell rosetting and emperipolesis**. Primary FDC-like cells at day 14 were co-cultured with B-cells (Raji) at a ratio of 1:1 for 4 hours, fixed and double-stained for AP and CD22. **a**, AP^pos ^FDC-like cells (blue) with filamentous dendrites capturing CD22^+ ^Raji cells (brown); **b**, survey of rosettes and various stages of emperipolesis.

When cultured for 14 days or longer, spontaneous homotypic clustering, B cell rosetting and emperipolesis, as well as antigen retaining in its native form (see below) were verifiable as characteristic functional features of FDCs. For these experiments, initially autologous B cells (not shown) and thereafter the Raji B-cell line was employed (cf. Fig. 5a, and 5b for B-cell rosetting and emperipolesis by FDC-like cells).

### Antigen trapping and retention

Antigen retention for a longer period of time may be regarded as the most defining and distinctive function of FDCs. In order to investigate whether cultured monocyte derivatives express this property, we employed horseradish peroxidase (HRPO) as a model antigen which offers the advantage to be easily detectable by a standard HRPO substrate reaction. The enzyme was offered in the form of immune complexes of human Ig/HRPO-anti-human IgG.

**Figure 6:**
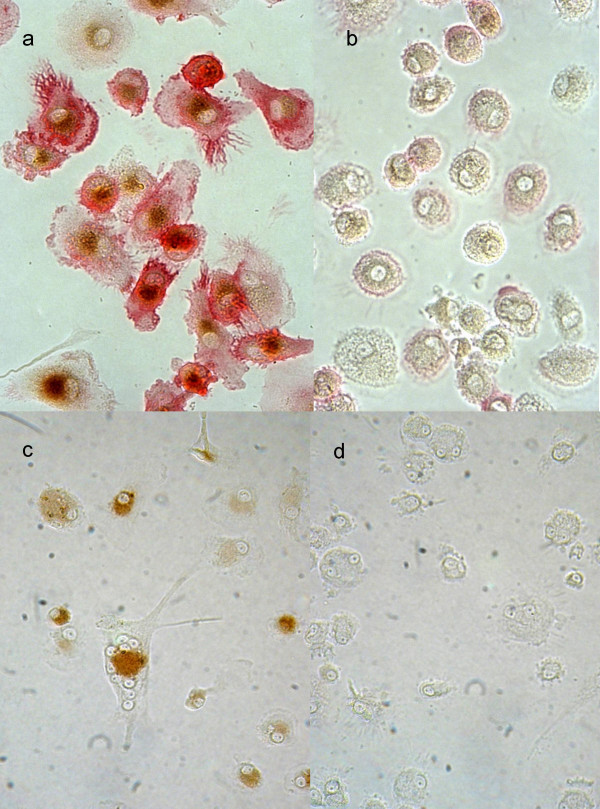
**Antigen trapping and long-term retention**. FDC-like cells (**a, c**) or macrophages (**b, d**), were pulsed for 30 min with HRPO/human IgG complexes. **a**, **b**, Cells were fixed after 4 days and double stained by a standard HRPO substrate reaction (brown) and for CD35 (red). **a**, In FDC-like cells antigen was present in its enzymatic active form, and CD35 was expressed. **b**, In macrophages only traces of antigen was detectable and CD35 is weakly positive. **c**, **d**, Cells were cultured for 16 days. In FDC-like cells (c) enzyme activity is still detectable, whereas in macrophages enzyme activity is absent.

Complexes were likewise trapped by monocyte-derived FDC-like cells and macrophages. In FDC-like cells HRPO remained fully enzymatically active up to 16-days tested (Fig. 6a, c). In contrast, control macrophages had almost completely degraded the enzyme at 4 and 16 days, respectively (Fig. 6b, d), already observable after 4 h (not shown). Immune-complex loaded cells strongly co-expressed CD35 as a typical FDC marker (Fig. 6c), whereas control macrophages only marginally expressed CD35. Only minimal numbers of cells revealed HRPO activity (Fig. 6d).

### Secondary stimulation

**Figure 7:**
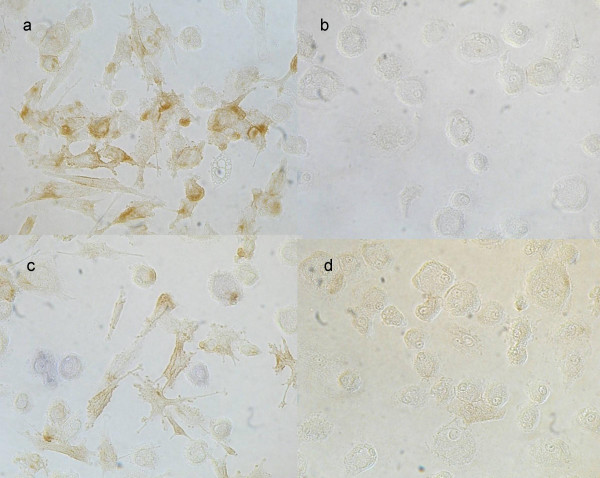
**Up-regulation of adhesion molecules after secondary stimulation**. TNF-α added to FDC-like cells on day 13 for further three days expressed ICAM-1 (CD54) (**a**) and VCAM (CD106) (**c**) on day 16; **b**, **d**, macrophages as controls.

Based on these findings, an even more mature FDC phenotype was differentiable from the AP^pos ^phenotype under secondary stimulation. When day12 AP^pos ^FDC-like cells were pulsed for a further 3 to 5 days with 10 ng/ml TNF-α, they were induced for increased expression of ICAM-1 (CD54) (Fig. 7a) and VCAM (CD106) was slightly up-regulated (Fig. 7c; see also Table 1). In control macrophage cultures these markers were seen only occasionally (Fig. 7b,d). Both adhesion molecules as well as CD35 are hallmarks of functionally competent FDCs *in vivo *[15,35]. Supplementing the cultures with TNF-β (LT-α) [36,37] did not reveal similar effects and IFN-γ up-regulated CD54 only, but down-regulated CD35 (not shown). Functional markers such as B-cell rosetting and antigen retention were not further up-regulated by secondary stimulation (not shown).

**Table 1:** Phenotype and function of primary and secondary FDC-like cells as compared with macrophages generated in vitro and with freshly prepared monocytes

	Monocytes [day 0]	Macrophages [day 15]	Primary FDC-like cells [day 15]	Secondary FDC-like cells [day 18]
HLA-DR	+	++	+	++
CD22	-	-	±	±
CD35 (CR1)	+	+	+++	+++
CD45	+++	+++	±	N.D.
CD54 (ICAM-1)	+	-	-	++
CD68	++	+++	+±	+±
CD106 (VCAM-1)	-	-	-	+
AP	-	-	+++	+++
Clustering.	-	-	++	+++
B cell Rosetting	-	-	+++	+++
Emperipolesis	-	-	++	++
Antigen Retention	-	-	+++	+++

Markers and functions are expressed as absent (-), faintly or few positive cells (+), positive with different intensities (+, ++, +++), variable with respect to donor variations (±). The term, clustering, refers to the ability to establish homotypic and heterotypic aggregates. N.D. not determined.

### Monocyte proliferation

Throughout the entire period of culture, the differentiating monocyte derivatives were tested in serial two day-intervals for 5-bromodeoxyuridine (BrdU) incorporation. The maximum of 1% positive cells was measured within days 6 to 10 (not shown). This result clearly excluded the outgrowth of a certain cell subset(s) while underscoring a direct derivation of AP^pos ^cells from monocytes.

As an alternative source, we used monocytes derived from 8- to 10-day cultures in which – as a first step – proliferation had been induced with GM-CSF at 25 U/ml. Cells transferred from this condition, and immediately introduced to FDC-inducing conditions specified above, ceased to proliferate and acquired the complete phenotype of primary FDC-like cells (not shown).

## Discussion

In the present study, we demonstrate for the first time the *in-vitro *generation of FDC-like cells from monocytes as their putative precursors. Human monocytes were prepared by selective adherence combined with reverse sedimentation as a novel procedure in order to eliminate undesired non-adherent cell populations. This protocol allowed for a virtual enrichment of monocytes while avoiding any interference with antibodies posing the potential hazard to evoke adverse cellular trigger events.

Our data clearly demonstrate that peripheral blood monocytes can be induced to differentiatiate into FDC-like cells. Similar to the earlier conundrum on the ontogeny of T cell-tropic dendritic cells (DCs) [21], the search for the origin of FDCs has always been complicated by their unstable marker profile, showing that FDCs isolated from lymphoid organs readily lose typical antigens such as DRC-1, CD21, CD23, and KiM4 in culture [35,38,39]. For practical reasons, we therefore decided to take advantage of an AP isoenzyme that has been described to be expressed by FDCs [30], but not by monocytes, macrophages or monocyte-derived DCs (cf. Tab. 1). Actually, our earlier experiments had revealed that this anchor marker is inducible in monocytes under defined conditions, suggesting AP expression as indicative for the developmental plasticity of these cells [27]. As a logical step, when attempting to generate FDCs *in vitro*, we first converted monocytes into AP^pos ^cells, here referred to as primary FDC-like cells.

Typically, the common leukocyte antigen CD45 was strongly down-regulated in AP^pos ^cells as well as in cells with similar morphology suggesting a transdifferentiation into non-leukocytic cells. Absence of CD45 has been described for FDCs by Schriever et al. [40]. The co-expression of CD68, however, indicates the monocytic origin of the AP^pos ^cells.

Labelling of CD35 (complement receptor I) is routinely used in histology to discriminate FDCs from other types of dendritic cells as well as from histiocytes [31,32]. In our work CD35 was stably expressed in monocyte-derived FDC-like cells. Again, we found the monocyte/macrophage marker CD68 in CD35^pos ^cells which was also obvious in CD35/AP co-expressing cells. Control macrophages were stained only marginally or negatively for CD35.

When establishing conditions for the generation of AP^pos ^cells, sera were found to give variable results. Therefore, we developed a completely serum-free differentiation protocol. Low concentrations of IL-4 and GM-CSF plus Dex were found to act as basic inducers. Within 12 to 15 days of culture, these conditions elicited a cell showing markers and functions of FDCs. Conversely, this combination of factors prevented the differentiation of macrophages or T-cell tropic dendritic cells from monocytes.

IL-4 at concentrations one order of magnitude lower than those established for the differentiation of T-cell tropic DCs had previously been shown to generate large flattened and adherent cells with fine dendritic protrusions. Such cells had shown to express both, AP as well as the myeloid marker CD68 [27]. Our forthcoming experiments revealed that IL-4 can successfully be replaced by IL-13, which will be a subject of further research.

Importantly, corticosteroids such as Dex synergistically enhanced AP expression. Dex, previously employed to induce osteoblasts from bone-marrow derived mesenchymal stem cells [41], has been shown to inhibit macrophage and DC differentiation [42]. When differentiating FDCs from monocytes, we now have found that IL-4 and Dex synergize in inducing the AP^pos ^phenotype.

GM-CSF which was applied routinely at low concentrations appeared merely to serve as a survival factor in addition to its contribution to differentiation. Its actual requirement was largely donor-dependent, indicating that developmental properties of starter monocytes may vary according to the donor's immunological status. Moreover, we have experienced that occasionally exogenous GM-CSF can even be omitted without affecting the differentiation towards AP^pos ^cells, which might be explained by an autocrine production of GM-CSF by monocytes in culture [43].

After 12–15 days of culture, the majority of monocyte-derived cells displayed the AP^pos ^phenotype, while not proliferating throughout this period of time. This high percentage of AP^pos ^cells therefore reflects quantitative monocytic differentiation and clearly argues against the possibility that FDC-like cells might have expanded from a small contaminating cell population.

Alternatively, we used monocytes in which – as a first step – proliferation had been induced by adding GM-CSF at higher concentrations. Cells transferred from this condition and introduced to FDC-inducing conditions acquired the AP^pos ^phenotype as well. As a consequence, combining sequential proliferation and differentiation might emerge as the method of choice when considering large-scale production of FDC-like cells from monocytes, for example when faced with the need to avail such cells for therapeutic application.

Membrane expression of ICAM-I and VCAM on FDCs has been described as a prerequisite for the interaction of FDCs and B cells within lymphoid tissues. We found a strong up-regulation of ICAM-I after secondary stimulation with TNF-α. However, we already obtained functionally competent FDC-like cells capable of B-cell rosetting and antigen retention which strongly expressed CD35 under the TNF-α-free basic condition.

Development of functional germinal centres as well as the maintenance of FDC function has been claimed to strongly depend on the engagement of TNF family members (TNF-α and LT-α/β) [36,37]. In-vitro generation of FDC-like cells, as described herein, appears to be widely independent of LT-α/β, whereas TNF-α may cause a further maturation of the FDC phenotype. The expression of HLA-DR which is controversial, but positively found by some authors [35,39], was up-regulated by TNF-α as well (Table 1).

Other factors were, however, inhibitory. Lymphocytes had to be eliminated as far as possible – which, especially when activated, inhibited the observed transdifferentiation. Specifically, IFN-γ may be the main factor: when added to monocytes as early as at the onset of culture, it strictly inhibited AP^pos ^phenotype, as we have shown before [27]. When added in a secondary step after 12 days of culture, IFN-γ up-regulated expression of CD54, but down-regulated CD35.

Of note, emperipolesis specifying the prolonged engulfment of B cells is a unique FDC property which, within germinal centres, is implicated in the protection of transiently internalised B cells from apoptosis [33]. It is envisioned that this property may be useful for preserving immunogenic or tolerogenic B cells triggered *in vitro *for their subsequent clinical application.

Identifying these newly differentiated cells as FDC-like cells culminates in the demonstration of storing antigen for long periods of time in its native form. This ability has been described as a unique feature of FDCs [34] but, to our knowledge, neither for other types of dendritic cells nor for macrophages. Specifically, in FDC-like cells the antigen, provided as HRPO-IgG complexes, was retained in an enzymatic active form up to 16 days tested. Enzyme activity was localized perinuclearly. In contrast, macrophages eliminated the material within a few hours. Further studies will reveal the precise kinetics of uptake, sub-cellular distribution, storage and possible re-expression on the FDC surface by ultrastructural investigations.

Monocyte-derived FDC-like cells can now be generated *in vitro*. Obviously, one pertinent employment of this system is to spur on the further elucidation of the underpinnings of immunological B-cell memory. Furthermore, cultures of FDCs may greatly facilitate research on the mechanisms underlying the capture and functional preservation of antigen. Next, because antigen retention is known to be exploited by pathogenic entities such as the human immunodeficiency virus-1 (HIV-1) [44] as well as prions [45], close investigation of these processes *in vitro *provides hope for important progress in these areas. For example, Smith *et al. *nicely demonstrated on isolated human FDCs, and in murine models alike, that FDC-entrapped HIV-1 remains highly infective for prolonged times, which appears to be due to protection of this virus from degradation [46]. Last but not least, this novel protocol might even foreshadow later clinical applications of autologous FDCs or FDC-preserved B cells akin to the clinical studies currently conducted with T cell-tropic DCs. In conclusion, the present findings may open up new horizons for unraveling the secrets of antigen and pathogen storage by FDCs, with self-evident immunological and clinical implications.

Other groups have shown other cell types to transdifferentiate from monocytic precursors. However, all of these studies suffered from the lack of defined conditions (such as by using serum, conditioned media or undefined lymphocyte-derived signals) and/or employed a monocyte subfraction as the starter population. In contrast, we now introduce a common pathway, and define the signals required, for allowing monocytes to quantitatively convert into a state of high developmental plasticity from which FDC-like cells can be deduced. It needs to be stressed that our results do not *per se *give a definitive clue as to the normal pathway of FDC differentiation. In fact, contemplating the actual existence of such a principal differentiation path may either unravel the main pathway of FDC generation or a salvage route, respectively. Normal FDCs *in situ *express some myeloid markers such as CD14 and CD11b but not CD68. However, FDC tumors have been described to also express CD68 [47-49]. In any case, the myeloid pathway desribed here obviously complements our previous findings on the ontogeny of T cell-directed DCs in that monocytes [20] and their myeloid precursors [50] can both be induced to quantitatively develop into T cell-tropic DCs, as is now commonly acknowledged.

Interestingly, AP and CD35 expression as well as antigen capture were occasionally observed to spontaneously emerge in single cells within control macrophage cultures. It thus becomes increasingly obvious that monocytes, macrophages, T-cell tropic DCs and FDCs belong to a continuum of a developmentally plastic system of cells which are highly interconvertible. Further data expand this view to osteoblasts, which were recently differentiated from a monocyte subset [24], thus supporting our results on overlapping phenotypes between osteoclasts, osteoblasts, dendritic cells and macrophages [27].

Moreover, as mentioned before, even cell classes generally considered to be developmentally distant from the myeloid lineage can be readily obtained from monocytes when the appropriate signals are provided. Hence, when acknowledging the monocyte as a previously ignored species of somatic stem cell, it appears unlikely that this fascinating cell type has already disclosed its entire potential. It will indeed be exciting to witness which ontogenetic surprises and therapeutic promises the future may hold.

## Conclusion

Here we provide evidence that monocytes can be transdifferentiated *in vitro *into cells that resemble FDCs by several markers and functions. Immunocytochemical co-stainings suggest thatthese cells had a myeloid origin. An attractive feature of our findings isthat theyprecisely describe stimuli enabling a transdifferentiation of monocytes into FDC-like cells at defined serum-free conditions. As a result, FDC-like cells can now be quantitatively produced *in vitro *for the first time, thereby offering a broad application spectrum ranging from basic research to clinical employment.

## Methods

### Monocyte isolation and culture

Leukocytes were obtained from leukapheresis of healthy blood donors, in addition with serum supplied by the blood transfusion service, University Hospital, Goettingen. Mononuclear cells were prepared by standard Ficoll-Hypaque (Nycomed, Oslo, Norway) gradient sedimentation, diluted with PBS to a final density of 1.068, resulting in an enrichment of monocytes of about 70%, and washed free of platelets. In some experiments, monocytes were obtained from counterflow elutriation (Elutra, Gambro, Martinsried, Germany). Cells were seeded into microwells at 30.000 monocytes/well, in the presence of 10% of 0.45 μm filtered human pooled serum and allowed to attach for a one hour. In order to obtain highly purified monocytes non-attached cells were separated from monocytes by a hanging-drop technique here referred to as reverse sedimentation: Flat-bottom wells were filled up with culture medium to form a convex meniscus, the microplates were then gently turned upside-down and cultured for 2 h in the inverted position. The medium containing non-attached cells was snicked off, and the adherent cells of about 95% purity (FACS analysis of CD14 positive cells, not demonstrated) were further cultured in 100 μl of differentiation medium: CellGro serum free medium (CellGenix, Freiburg, Germany) supplemented with N-acetyl-L-alanyl-L-glutamine (Biochrom, Berlin, Germany), penicillin/streptomycin (Biochrom), 10^-6 ^M dexamethasone dinatrium phosphate (Ratiopharm, Ulm, Germany), 25 U/ml recombinant human Il-4, 3 U/ml recombinant human GM-CSF (R&D Systems, *Wiesbaden*, Germany). Lymphocytes, if still present, were additionally washed away on the second day. Once a week, medium plus additives were completely exchanged. During the entire cell preparation and culture care was taken to minimize evaporation and pH shift to alkaline. At day 15 the cultures were terminated or, alternatively, additional inducers (recombinant TNF-α, LT-α/β, IFN-γ (R&D Systems)) were added at day 13 for another three days. For control, macrophages were differentiated in medium consisting of each 50% "Medium 199" and RPMI (Biochrom) supplemented with penicillin/streptomycin and 10% heat-inactivated human serum (pooled from 30 healthy donors).

### Immunocytochemistry

For staining procedures cells were fixed in ethanol/acetic acid (95%/5%) for 30 min or alternatively with cold methanol for 5 min and rehydrated by 3× washing in distilled water. Antibodies (from Dako, Glowstrup, Denmark, if not stated otherwise) were diluted in medium plus 10% human sera: anti-CD68 clone EMB11 1:100, anti-human CD35 (FDC) 1:30, anti-human CD54 1:800, anti-human CD106 1:30, anti-human HLA-DR 1:200, anti-human CD45 1:100, anti-human CD22 1:50, AP-anti-human IgM 1:50, goat-AP-anti-mouse IgG 1:30, anti-BrdU, (Amersham, Braunschweig, Germany) undiluted, sheep-PO-anti mouse IgG, (Amersham) 1:300. The primary antibodies were incubated over night at 4°C, the secondary antibodies at RT for 45 min. After each incubation step the preparations were washed with PBS. The PO-staining was developed with H_2_O_2 _(1 μl/5 ml) and diaminobenzidine (50 μg/ml) in PBS/distilled water 1:1. The AP-conjugated secondary antibodies were detected with Naphthol/Fast Red, Sigma Deisenhofen, Germany, including levamisole to inhibit edogenous AP activity. Staining controls were done by omitting the primary antibodies. The substrate reactions were observed microscopically and stopped with PBS. The preparations were stored in PBS/glycerine 1:1 at 20°C. For immunodoublestaining the preparation after the first staining procedure was washed extensively with PBS, the next immunostaining was performed using a differently conjugated secondary antibody.

### Enzyme cytochemistry for AP

The enzymecytochemical detection of AP was performed using a test kit from Sigma according to the manufactures instructions with the following modification: The cultures were fixed as described for immunocytochemistry. The enzyme reaction was carried out at RT for 20 min.

### Enzyme cytochemical/immunocytochemical double or triple staining

Enzymecytochemical staining was performed prior to immunostaining. After enzymestaining the preparation was washed 3× in distilled water, the subsequent immunostaining was performed as described above. For further immunostaining the preparation was washed again extensively before the next immunostaining was performed using a differently conjugated secondary antibody.

### Cell proliferation assay

Cell proliferation was determined by incorporation of BrdU for 24 h into the nuclei follwed by immunostaining with PO using a test kit from Amersham according to the manufacturer's instructions.

### Immunoglobulin aggregation

In order obtain aggregated immunoglobulines a purified immunoglobulin preparation (Polyglobin, Behringwerke, 10% Ig in physiologic saline) was used at 10% in PBS and incubated for 60 min at 60°C.

### B-cell rosetting and emperipolesis

Raji cells (human B cell line) were used as a source of B lymphocytes. For demonstrating rosetting or emperipolesis, the Raji cells were cocultured at **3 × 10^4 ^**cells/microwell with the monocyte-derived cells. After **4 **hours the cultures were fixed with ethanol/acetic acid stained, as above and evaluated microscopically.

### Antigen trapping

Horseradish peroxidase (Calbiochem, Bad Soden/Ts, Germany) was employed as a model antigen. The enzyme was offered in the form of immune complexes of human Ig (Behringwerke Marburg, Germany), HRPO-anti-human IgG (goat) plus HRPO-anti-goat IgG for enhancing the reaction. Immune complexes were added at 10% on day 12 to 15 for 30 min, thereafter the medium was changed. After further incubation up to 16 days the cultures were fixed with methanol for 5 min. and stained for peroxidase as described for immunocytochemistry and reacted for about 10–15 min. The reaction was stopped by washing with distilled water.

## Authors' contributions

DH has proposed the AP^pos ^phenotype to be the link to FDCs. She has established the main protocol for generation of AP^pos ^cells from monocytes as well as most of the experiments. JHP has developed the novel described monocyte purification, introduced the use of Il-13 as equivalent to Il-4. He contributed to the theoretical framework.
